# Exercise-Based Prehabilitation Before Cardiac Surgery: A Systematic Review, Meta-Analysis, Meta-Regression, and Proposal for a Clinical Implementation Model

**DOI:** 10.3390/jcm14228195

**Published:** 2025-11-19

**Authors:** Juan Carlos Hurtado-Borrego, Adrián Bayonas-Ruiz, Bárbara Bonacasa

**Affiliations:** 1 Physical Medicine and Rehabilitation Service, Virgen de la Arrixaca University Hospital, El Palmar, 30120 Murcia, Spain; juancarlos.hurtadob@um.es; 2 Department of Physiology, Human Physiology Area, Edificio LAIB/Departamental, Campus CC Salud-El Palmar, Campus de Excelencia Internacional de la Universidad de Murcia, 30120 Murcia, Spain; adrian.bayonas@um.es; 3 Research Group of Physical Exercise and Human Performance, Edificio LAIB/Departamental, El Palmar, Campus de Excelencia Internacional de la Universidad de Murcia, 30120 Murcia, Spain

**Keywords:** preoperative exercise, preoperative care [MeSH], exercise therapy [MeSH], heart surgical procedures [MeSH], rehabilitation [MeSH], exercise capacity [MeSH], sarcopenia [MeSH]

## Abstract

**Background/Objectives**: Major cardiac surgery is associated with a high rate of postoperative complications, particularly in older and frail patients. Prehabilitation—defined as a preoperative intervention based on structured exercise—aims to enhance patients’ physiological and functional reserve before surgery. To evaluate the effectiveness of prehabilitation programs on functional capacity and postoperative complications in cardiac surgery and to propose a clinical exercise-based intervention model tailored to these patients. **Methods**: A systematic search was conducted in PubMed, Cochrane, PEDro, and LILACS (2005–2025). Randomized controlled trials investigating preoperative exercise interventions in adults undergoing cardiac surgery were included. Outcomes assessed included functional measures (6-Minute Walk Test [6MWT], Timed Up and Go test [TUG], maximal oxygen uptake [VO_2_max], maximal inspiratory pressure [MIP]), frailty (Clinical Frailty Scale [CFS], Essential Frailty Toolset [EFT]), postoperative complications and quality of life. **Results**: Nine studies comprising a total of 873 patients were included. Prehabilitation significantly improved functional capacity (∆6MWT: +52.4 m; *p* < 0.001), reduced respiratory complications (pneumonia, atelectasis) and shortened hospital stay (−15.2 h; *p* < 0.001). The greatest benefits were observed in multimodal programs lasting ≥4 weeks. **Conclusions**: Exercise-based prehabilitation is an effective and safe strategy in patients awaiting cardiac surgery. Its systematic implementation should be considered as part of the perioperative pathway, reinforcing the role of exercise as a therapeutic tool in this clinical context.

## 1. Introduction

Major cardiac surgery, such as coronary artery bypass grafting or valve replacement, imposes substantial physiological stress, particularly in patients with frailty, multiple comorbidities and limited functional capacity. Current evidence supports prehabilitation as an effective intervention to enhance physiological resilience before surgery by improving cardiovascular, pulmonary, and musculoskeletal reserve. This strategy has been primarily studied in patients with cancer [1].

Even short periods of tailored training can induce meaningful improvements in maximal oxygen uptake (VO_2_max), muscle strength, exercise tolerance and respiratory function. In this context, prehabilitation is defined as a multimodal intervention, mainly exercise-based, designed to optimize patients’ functional status prior to surgery [2]. From a physical and rehabilitation medicine perspective, its relevance lies in the ability to reduce complications, accelerate recovery and facilitate functional reintegration, especially in patients with low aerobic capacity (<14 mL/kg/min), which has been associated with a higher risk of adverse postoperative outcomes [3].

Nevertheless, there is considerable heterogeneity in published prehabilitation protocols for cardiac surgery patients regarding duration, intensity, training volume, modalities and assessment tools. In addition, frailty, sarcopenia and dynapenia remain underrecognized as therapeutic targets.

Therefore, the main aim of this study was to synthesize the available evidence on the effectiveness of prehabilitation programs in cardiac surgery, assessing their impact on functional capacity, frailty, postoperative complications and quality of life, as well as to propose an exercise-based clinical model for implementation in routine practice.

## 2. Materials and Methods

This systematic review was conducted in accordance with the PRISMA 2020 statement and the methodological principles of the Campbell Collaboration, and the completed checklist is provided in the Supplementary Table S1. The protocol was prospectively registered (PROSPERO; ID CRD420250495373) in September 2025.

### 2.1. Search Strategy, Inclusion and Exclusion Criteria

A systematic search was performed in March 2025 in PubMed, Cochrane Library, PEDro, and LILACS to ensure comprehensive retrieval of studies from both Anglophone and non-Anglophone sources, covering the period 2005–2025. The search combined terms such as “prehabilitation,” “preoperative exercise,” “cardiac surgery,” “valvular surgery,” “coronary artery bypass graft,” “functional capacity,” and “exercise capacity.” The complete PubMed search strategy was: (((((prehabilitation) OR (preoperative exercise)) AND (cardiac surgery)) OR (valv*) OR (coronary artery bypass graft surgery) AND (functional capacity) NOT (transcatheter))) NOT (cancer). Filters: randomized controlled trial; publication date from 2005 to 2025. The full electronic search strategies for all databases are provided in Supplementary Table S2 Additional studies were identified through reference lists of previous reviews.

Inclusion criteria comprised randomized clinical trials of prehabilitation interventions delivered prior to cardiac surgery in adult patients (≥18 years), published in peer-reviewed journals in either English or Spanish and involving participants with a cardiac condition requiring surgical treatment. Conversely, exclusion criteria encompassed non-randomized clinical trials, prehabilitation programs lasting fewer than 5 days or studies in which the surgical procedure was not cardiac surgery.

Two independent reviewers (JCH, BB) screened titles/abstracts, retrieved full texts for potentially eligible articles and resolved discrepancies by consensus or consultation with a third reviewer (AB). Inter-reviewer agreement was assessed using the percentage of agreement for study selection and risk of bias assessment. The selection process was documented in a PRISMA flow diagram.

### 2.2. Data Extraction

Data were independently extracted using standardized forms and entered into Microsoft Excel^®^. Extracted variables included study characteristics (year, country, sample size, type of surgery), patient demographics, comorbidities, details of the prehabilitation intervention (duration, intensity, modality), assessment tools (functional tests, frailty scales, quality-of-life questionnaires) and postoperative complications, which were defined variably across studies. Most trials focused on pulmonary events such as atelectasis or prolonged mechanical ventilation, whereas others additionally reported cardiac or infectious complications. To ensure comparability across studies, durations of ICU and hospital stay were converted to hours as appropriate, and only outcomes reported with compatible metrics were quantitatively synthesized. When different instruments were used to assess functional capacity or quality of life, results were summarized narratively.

### 2.3. Risk of Bias Assessment

Publication bias was assessed through visual inspection of funnel plots (Supplementary Figure S1). The internal validity of the included studies was assessed using the Cochrane Risk of Bias tool (RoB 2) (see Supplementary Table S3). Disagreements were resolved by consensus (JCH, BB) or by consulting a third reviewer (AB).

### 2.4. Quality of Evidence Assessment

The certainty of the evidence for each outcome was assessed using the GRADE (Grading of Recommendations Assessment, Development and Evaluation) approach. The evidence was downgraded or upgraded based on the domains of risk of bias, inconsistency, indirectness, imprecision, and publication bias as proposed by the Cochrane Group. Evidence profiles and Summary of Findings tables were generated using a custom Word file created ad hoc that can be consulted in the Supplementary Table S4.

### 2.5. Statistical Analysis

Random-effects meta-analyses were performed using Review Manager (RevMan v5.4). For continuous variables (6-Minute Walk Test [6MWT], hospital stay, ICU stay, time to extubation), pooled effects were expressed as mean difference (MD) with 95% confidence intervals (CI). For dichotomous outcomes (arrhythmias, atelectasis, pneumonia), odds ratios (OR) with 95% CI were calculated. Statistical heterogeneity was assessed with χ^2^ and I^2^ statistics, considering *p* < 0.05 significant. To further explore heterogeneity, subgroup analyses were performed for the 6MWT outcome according to program duration (≤4 vs. >4 weeks) and inclusion of inspiratory muscle training. Publication bias was explored by visual inspection of funnel plots.

#### 2.5.1. Meta-Regression

To further explore potential sources of heterogeneity in the pooled results, we conducted univariable random-effects meta-regression analyses using SPSS v30 (IBM Corp., Armonk, NY, USA). The dependent variable was the MD in 6MWT between intervention and control groups. Three potential covariates that could influence the improvement in functional capacity were selected, as these were the only variables consistently reported across studies: the duration of the prehabilitation program (weeks), the mean age of the patients (years) and the inclusion of respiratory training in the protocol.

#### 2.5.2. Subgroup Analyses

Subgroup analyses were performed in RevMan 5.4. Subgroup analyses were performed on changes in 6MWT distance with subgroups based on prehabilitation duration (≤4 weeks vs. more than 4 weeks) and inclusion of respiratory muscle training (yes vs. no).

#### 2.5.3. Sensitivity Analyses

Sensitivity analyses were carried out to assess the influence of individual studies on the overall meta-analysis estimates, using a leave-one-out approach, whereby the meta-analysis was repeated after sequentially excluding each individual study. A study was considered for exclusion if it met three predefined criteria: (i) its weight exceeded twice the expected average weight of the included studies; (ii) its presence altered the MD and 95% CI by more than 25%; and (iii) its inclusion changed the statistical significance of the results (from significant to non-significant or vice versa). This strategy ensured that studies exerting a disproportionately high influence on the pooled estimates were appropriately addressed.

## 3. Results

### 3.1. Study Characteristics

**Figure 1:**
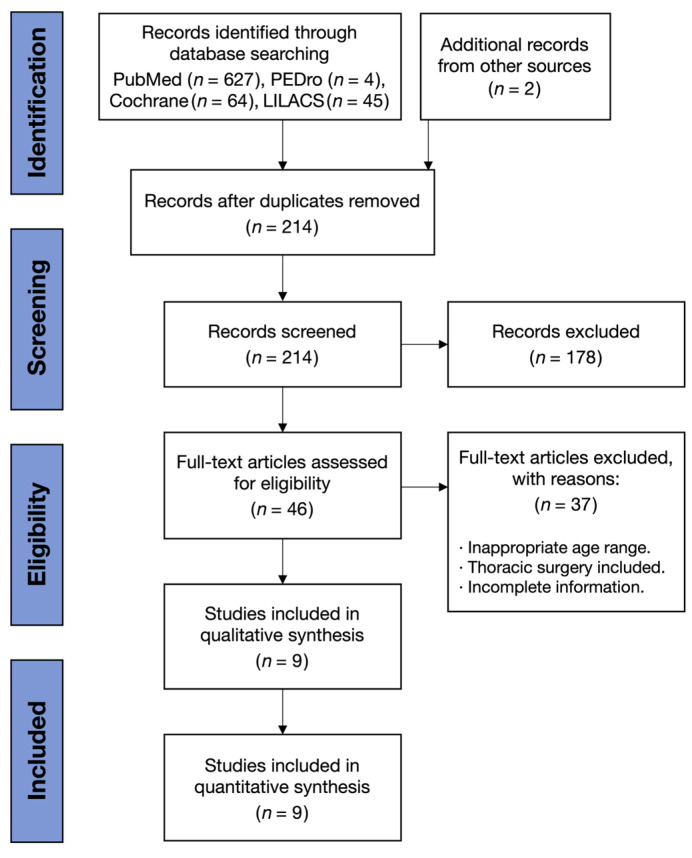
PRISMA flow chart of the search.

A total of 327 records were identified through database searching. After removing duplicates, 214 titles and abstracts were screened, of which 178 were excluded because they did not involve preoperative exercise interventions, were non-randomized or included only postoperative rehabilitation programs. 46 full-text articles were assessed for eligibility, and 37 were excluded for reasons including not being related to cardiac surgery (n = 18), multimodal interventions not isolating exercise effects (n = 8) and insufficient outcome data (n = 11). Finally, nine randomized controlled trials [4,5,6,7,8,9,10,11,12] that included 873 patients were included in the quantitative synthesis (Figure 1) [13].

Agreement between reviewers was 100% for study selection and risk of bias assessment. Overall methodological quality was moderate. Five studies were rated as low risk of bias, three as having some concerns and one as high risk, mainly related to lack of blinding and incomplete outcome data (Supplementary Table S3). The specific details of each study are presented in Table 1. The studies were conducted in eight different countries between 2008 and 2025, with sample sizes ranging from 17 to 230 participants.

Populations were predominantly older adults (>65 years) and mostly male (≈75%). Most trials included patients undergoing coronary artery bypass grafting (CABG), while four also included valve surgery and one focused exclusively on valvular procedures. In all but one study, the control group received either no intervention or general lifestyle advice, whereas in Sahar et al. [11], the control arm was instructed in inspiratory muscle exercises. Prehabilitation programs varied considerably in structure and content. Program duration ranged from 2 to 8 weeks, with sessions typically performed 2 to 3 times per week. Most interventions combined aerobic and resistance training, while four trials also incorporated inspiratory muscle training. Exercise intensity was generally light, with aerobic sessions commonly performed on a cycle ergometer and resistance training targeting large muscle groups using elastic bands or light weights. The main characteristics and training parameters are summarized in Supplementary Table S5.

### 3.2. Characteristics of Prehabilitation Programs

The prehabilitation programs varied considerably across studies, ranging from 5 days to 8 weeks and combining aerobic, resistance and, in some cases, respiratory muscle training (Table 2). Aerobic exercise was delivered mainly on cycle ergometers or treadmills, while resistance training used elastic bands, dumbbells, or bodyweight. Inspiratory muscle training was included in four trials, although often poorly described. Intensity was generally light, defined by maximum heart rate (HRmax), maximal oxygen uptake or one-repetition maximum (1RM) when specified. Only Sawatzky et al. [6] applied moderate-intensity continuous training (MICT) and López-Hernández et al. [10] used high-intensity interval training (HIIT). Overall, the interventions lacked standardization in duration, intensity and exercise prescription, underscoring the need for a structured clinical model.

### 3.3. Functional Capacity

**Figure 2:**
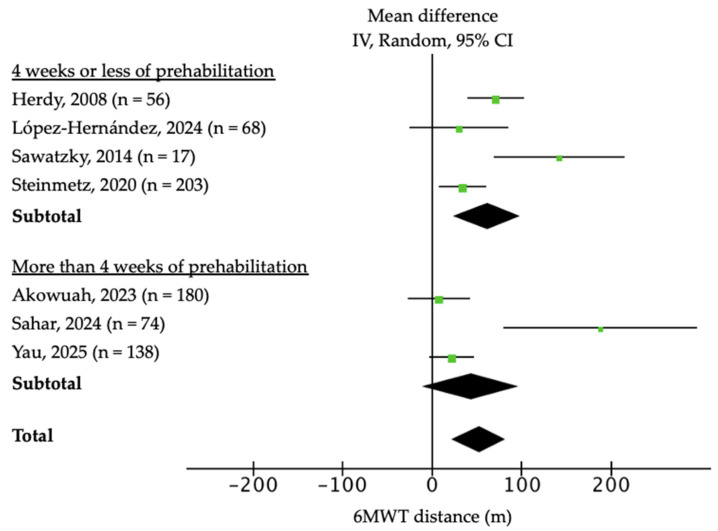
Forest plot of the effect of prehabilitation on 6 min walk test (6MWT) and subgroup analysis according to program duration [4,6,8,9,10,11,12].

**Figure 3:**
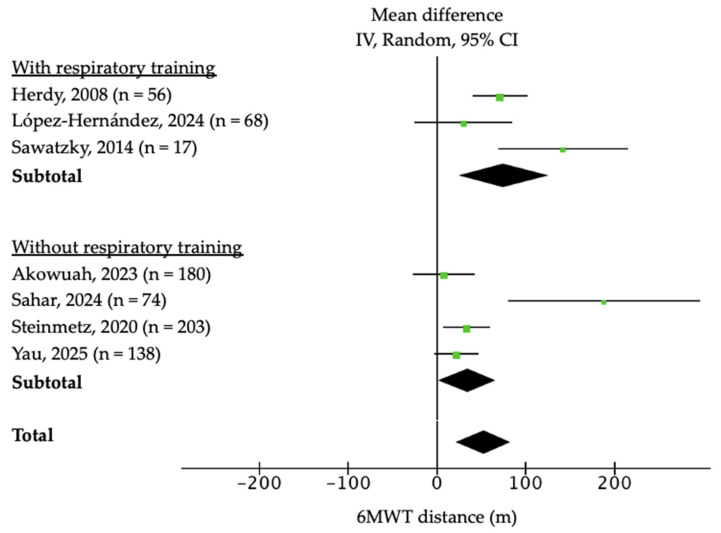
Forest plot of the effect of prehabilitation on 6 min walk test (6MWT) and subgroup analysis according to the inclusion (or not) of respiratory muscle training [4,6,8,9,10,11,12].

Functional capacity was mainly assessed using 6MWT, which was performed in eight of the nine trials. The meta-analysis showed a significant improvement of +52.4 m in the intervention group (n = 724; 95% CI: 22.4–82.1; *p* < 0.001; I^2^ = 76%; Figure 2) with a moderate certainty of the evidence according to the GRADE scale. Sensitivity analysis excluding Sahar et al. [11] and Sawatzky et al. [6] yielded a pooled MD of +46.2 m (95% CI: 27.8–64.6; *p* < 0.001), with heterogeneity decreasing from 76% to 42%. Sawatzky et al. [6] confirmed this benefit at 3 months post-surgery (+123 m, 95% CI: 62–209), and Steinmetz et al. [8] at 3 weeks (+39.1 m, 95% CI: 21–54). A considerable heterogeneity was found in the overall analysis (I^2^ = 76%). In subgroup analyses based on program duration (Figure 2) and inclusion of respiratory muscle training (Figure 3), heterogeneity decreased to 0% and 45.9%, respectively. Programs lasting ≤ 4 weeks showed larger mean gains in 6MWT distance (n = 344; MD: 61.31; 95% CI: 24.1 to 98.2; *p* < 0.001; I^2^ = 70%) compared with longer programs (n = 380; MD: 43.61; 95% CI: −10.42 to 97.1; *p* < 0.11; I^2^ = 80%), whereas interventions including inspiratory muscle training achieved improvements (n = 141; MD: 75.5; 95% CI: 25.3 to 125.5; *p* = 0.05; I^2^ = 66%) relative to those without respiratory training (n = 583; MD: 34-05; 95% CI: 1.56 to 66.54; *p* = 0.004; I^2^ = 76%).

**Figure 4:**
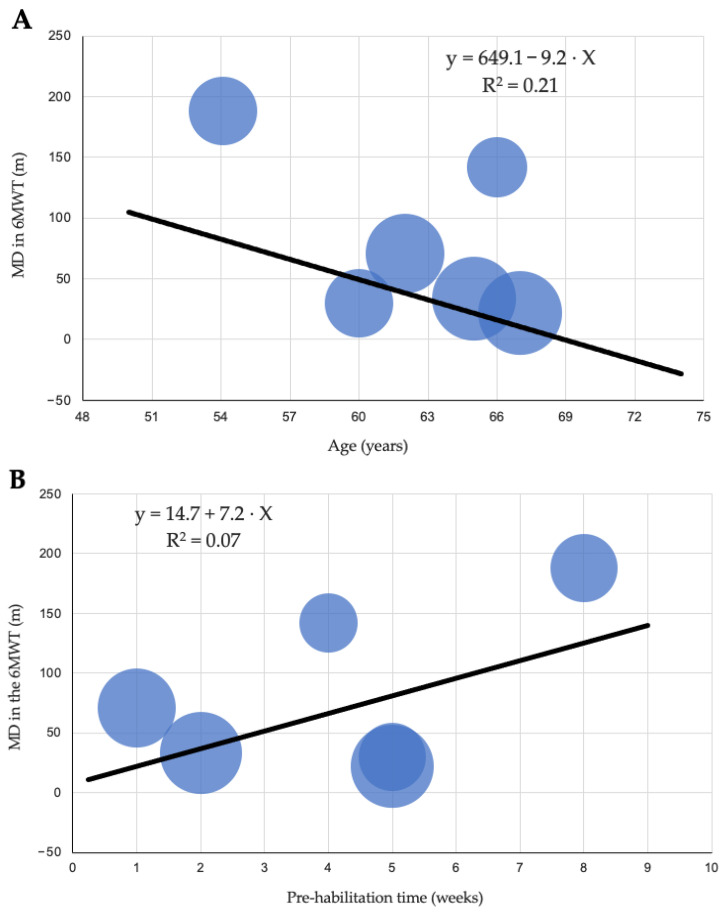

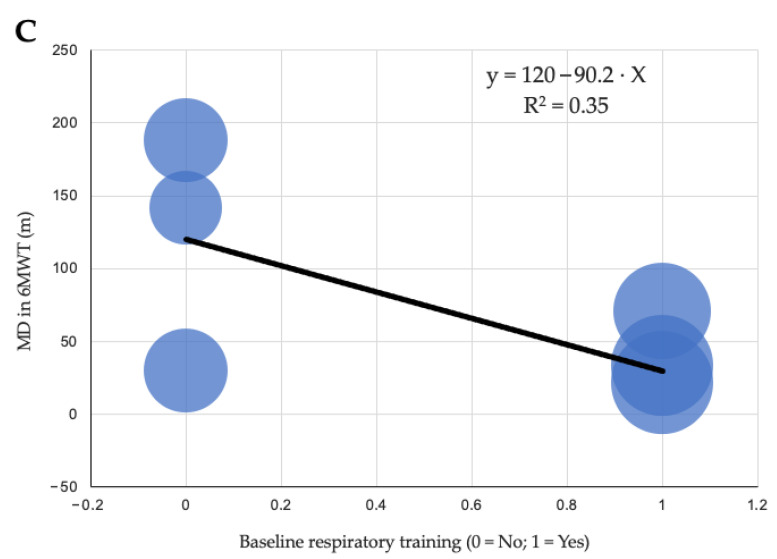
Meta-regression analysis of moderators influencing the effect of exercise-based prehabilitation on functional capacity as assessed by the 6 min walk test (6MWT): (**A**) age, (**B**) duration of prehabilitation, and (**C**) inspiratory muscle training.

Meta-regression analyses showed a positive but non-significant association between program duration and functional improvement (B = 14.8; *p* = 0.287; R^2^ = 0.22), an inverse trend with age (B = −9.3; *p* = 0.169; R^2^ = 0.34) and smaller gains in studies that incorporated respiratory training (B = −90.3; *p* = 0.094; R^2^ = 0.46). (Table 2, Figure 4).

### 3.4. Frailty

Frailty was assessed using validated scales. In Waite et al. [7] (n = 20), the Clinical Frailty Scale (CFS) score decreased significantly from 4.58 ± 0.96 to 4.05 ± 1.1 after prehabilitation, corresponding to an 11.6% reduction (*p* < 0.001). Similarly, Sahar et al. [11] (n = 74) reported a significant improvement in CFS, with scores decreasing from 4.21 ± 1.18 to 3.18 ± 0.39 in the intervention group, compared with 4.00 ± 1.37 to 3.48 ± 0.65 in the control group (*p* < 0.001). In the same trial, frailty assessed with the Essential Frailty Toolset (EFT) also improved significantly, with intervention participants reducing their score from 4.35 ± 0.58 to 1.18 ± 0.39, compared with 1.45 ± 0.55 in the control group (*p* < 0.001).

### 3.5. Sarcopenia and Dynapenia

Muscle strength and sarcopenia-related parameters were reported in a limited number of studies. Akowuah et al. [9] (n = 180) classified patients with low handgrip strength as “sarcopenic” and found that these individuals experienced greater functional improvement in 6MWT after prehabilitation (*p* = 0.004). Similarly, López-Hernández et al. [10] observed significant preoperative gains in handgrip strength (+2.9 kg; *p* < 0.05), although changes did not reach statistical significance in the overall pooled analysis.

### 3.6. Postoperative Complications

**Figure 5:**
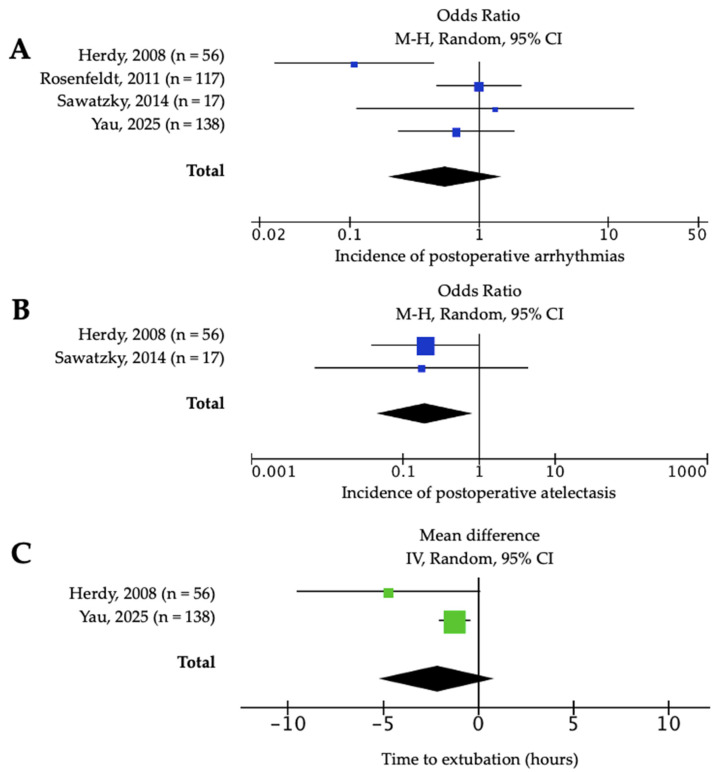
Forest plot of the effect of prehabilitation on early postoperative complications: (**A**) arrhythmias; (**B**) atelectasis; and (**C**) time to Extubation [4,5,6,12].

Postoperative complications were reported in most trials. Prehabilitation significantly reduced respiratory complications (Figure 5): the incidence of atelectasis was lower in the intervention group (n = 248; OR = 0.41, 95% CI: 0.18–0.91; *p* = 0.03; I^2^ = 0%, Figure 5B) with a high certainty of the evidence (Supplementary Table S4). No significant differences were observed in arrhythmias (n = 325; OR = 0.54, 95% CI: 0.20–1.49; *p* = 0.24; I^2^ = 62%, Figure 5A) or mortality (n = 523; OR = 0.67, 95% CI: 0.25–1.77; *p* = 0.42; I^2^ = 0%).

**Figure 6:**
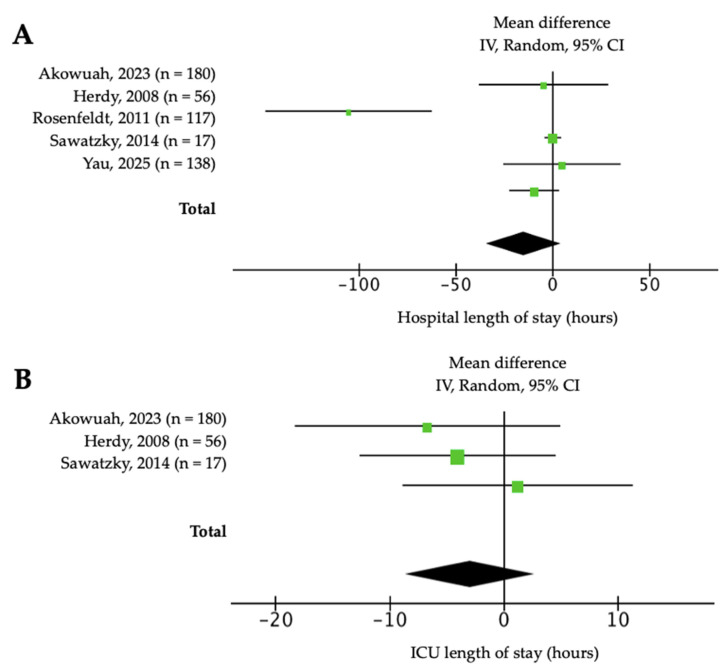
Forest plot of the effect of prehabilitation on the length of stay outcomes at (**A**) hospital; and (**B**) intense care unit (ICU) [4,5,6,9,12].

Regarding perioperative times, hospital stay was significantly shorter in the intervention group (n = 501; MD = −15.2 h, 95% CI: −34.6 to 4.22; *p* < 0.001; I^2^ = 84%, Figure 6A). For ICU stay duration, the pooled MD was −0.76 h (95% CI: −1.52 to −0.01; *p* = 0.05; I2 = 0%). Sensitivity analysis excluding Yau et al. [12] yielded −3.1 h (95% CI: −8.69 to 2.63; *p* = 0.57; I^2^ = 0%, Figure 6B), indicating that this large study had a substantial and disproportionate impact on the overall effect estimate, and therefore it was left out of the analysis. Extubation time was also not significantly different (n = 332; MD = −2.16 h, 95% CI: −5.16 to 0.84; *p* = 0.16; I^2^ = 50%). The certainty of the evidence in the latter outcomes ranged from very low to moderate (Supplementary Table S3). Other reported complications, such as pleural effusion, reoperation or stroke, did not differ between groups.

Yau et al. [12] additionally reported Days Alive and at Home at 30 days (DAH30) as a patient-centered outcome. No significant differences were observed between groups (n = 168; median 20.5 days in the intervention group vs. 21.5 in controls; *p* = 0.314).

### 3.7. Quality of Life

Quality of life was assessed using different patient-reported outcomes, including SF-36, EQ-5D, QoR-15, HADS, YPAS and DASI. Overall, no consistent improvements were observed across trials. SF-36 and EQ-5D scores showed non-significant changes in most domains. QoR-15 scores on postoperative day 3 did not differ significantly between groups (median difference −3, 95% CI −9 to 3; *p* = not specified) [12], although disability at 90 days measured by WHODAS 2.0 was significantly lower in the prehabilitation group (*p* = 0.022). No significant differences were observed in DASI or YPAS scores, whereas HADS scores showed only minimal changes [7].

## 4. Discussion

This systematic review with meta-analysis and meta-regression compiles the most recent evidence on prehabilitation in cardiac surgery. It provides an updated synthesis of current knowledge in this field. Prehabilitation was associated with improvements in functional capacity and a lower incidence of postoperative complications, particularly among frail patients or those with age-related skeletal muscle impairment.

This synthesis includes nine randomized controlled trials (873 participants), thus building upon prior systematic reviews that included 665 and 726 patients, respectively [14,15]. Most participants were older than 65 years, the age group most frequently undergoing cardiac surgery [16]. Approximately 80% of participants were male, with the lowest proportion being 69%, highlighting the underrepresentation of women. None of the included studies analyzed whether outcomes differed by sex.

Included programs varied widely in duration, intensity, and exercise prescription. Most trials focused on aerobic and resistance training, with inspiratory muscle training introduced as an adjunct in some protocols. However, very few studies provided detailed information on training progression, target muscle groups, or supervision, limiting reproducibility and comparability.

### 4.1. Functional Capacity

Our meta-analysis found that prehabilitation was associated with improved functional capacity, exceeding previously reported thresholds for clinical relevance in cardiac and pulmonary populations [17,18]. These gains were maintained at follow-up in some studies, underscoring the clinical relevance of preoperative exercise. The sensitivity analysis confirmed that the improvement in functional capacity was not driven by any single study. The reduction in heterogeneity after excluding Sahar et al. [11] and Sawatzky et al. [6] suggests that differences in program duration, training intensity and baseline frailty levels contributed to the observed variability rather than methodological inconsistencies. Overall heterogeneity decreased after introducing subgroups based on program duration and inclusion of respiratory muscle training. Shorter programs appeared to elicit greater short-term functional gains. This finding may reflect differences in program intensity and patient selection rather than true duration effects. Moreover, shorter interventions were typically more intensive and closely supervised, which may have optimized adherence and accelerated physiological adaptation. In contrast, trials including inspiratory muscle training showed greater improvements in 6MWT performance, likely reflecting the contribution of enhanced ventilatory efficiency and respiratory muscle endurance to global exercise tolerance. Additional functional tests, including TUG and gait speed, showed consistent improvements, while handgrip strength revealed only small, non-significant changes, suggesting that endurance and mobility outcomes are more responsive to short-term interventions. This lack of improvement in grip strength likely reflects inadequate prescription and the absence of detailed strength protocols. It highlights the importance of incorporating structured, individualized resistance training as a potentially key component of prehabilitation, given its close relationship with functional capacity and postoperative outcomes [19].

An important finding was that only López-Hernández et al. [10] evaluated functional capacity with CPET, reporting a significant increase in endurance time, which suggests that prehabilitation may also enhance global exercise tolerance, a key factor in withstanding surgical stress and facilitating recovery [3,20]. In parallel, inspiratory muscle training has proven effective in increasing MIP and reducing postoperative pulmonary complications. Its relevance was further underscored during the COVID-19 pandemic, where it improved respiratory strength, reduced dyspnea, increased 6MWT distance and enhanced quality of life in post-COVID patients [21]. Together, these findings support the role of respiratory training as an important element of multimodal prehabilitation and encourage its consideration in preoperative cardiac surgery programs.

Meta-regression analyses, although non-significant, indicated clinically meaningful trends: longer program duration was associated with greater gains, older age with smaller improvements and the inclusion of respiratory training with attenuated effects. The limited number of available trials may have contributed to the lack of statistical significance, and these relationships should continue to be examined as more studies emerge.

### 4.2. Frailty

Frailty is a key prognostic factor in cardiac surgery and highly prevalent in this population [22]. It was most frequently assessed with the CFS, sometimes complemented by the EFT. Our results suggest that prehabilitation may help reduce preoperative frailty, an important finding given its association with complications, longer hospital stay and increased perioperative mortality [23]. Nevertheless, several trials that assessed frailty did not report post-intervention comparisons, limiting conclusions. Despite its strong prognostic value, frailty was rarely assessed systematically. We consider frailty not only a risk marker but also a therapeutic target for prehabilitation, as exercise-based programs may reverse pre-frailty or frailty states and thereby improve outcomes.

### 4.3. Sarcopenia and Dynapenia

A largely underexplored aspect within prehabilitation research is dynapenia. In clinical practice, dynapenia (loss of strength without evident muscle mass reduction) is often confused with sarcopenia, although they are not equivalent [24,25]. Only Akowuah et al. [9] assessed muscle strength through handgrip testing, which primarily reflects dynapenia rather than sarcopenia. We believe it is important to highlight this distinction, as dynapenia itself is associated with poorer functional capacity, higher complication rates and slower recovery after cardiac surgery. To better identify vulnerable patients, the ISarco PRM protocol may serve as a useful framework for assessing muscle quality. It incorporates ultrasound-based muscle assessment using the Skeletal Muscle Thickness And Ratio (STAR) criterion, together with a multimodal intervention plan based on aerobic and resistance training and adequate nutritional support. Such strategies have been shown to improve muscle strength, mass, and functional capacity in relatively short periods [26,27,28] and could be particularly useful in patients awaiting cardiac surgery.

### 4.4. Postoperative Complications

Prehabilitation was associated with fewer respiratory complications, particularly atelectasis, which is among the most frequent and clinically relevant events after cardiac surgery [29] and may contribute to faster postoperative recovery. In contrast, no significant differences were observed in arrhythmias, ICU stay or mortality, outcomes likely influenced by their multifactorial nature and the limited number of trials reporting them. The heterogeneity of the included studies—differing in type of surgery, prehabilitation intensity, and preoperative protocols—likely influenced the magnitude of the effects, as illustrated by the high variability in hospital length of stay (I^2^ = 84%). Taken together, these findings underscore the clinical importance of incorporating respiratory training and aerobic exercise as essential components of multimodal prehabilitation.

### 4.5. Quality of Life

Patient-reported outcomes such as SF-36, EQ-5D, QoR-15, HADS and DASI were inconsistently affected by prehabilitation. A particularly relevant finding was that patients engaged in prehabilitation showed greater adherence to exercise and a significant increase in habitual physical activity, as reflected by YPAS scores. This behavioral change is clinically relevant, as regular physical activity improves functional capacity, reduces cardiovascular risk and enhances long-term well-being [30]. Therefore, even in the absence of immediate changes in most quality-of-life scales, the increased predisposition to maintain physical activity should be regarded as a positive outcome with potentially meaningful effects on prognosis and future quality of life.

Overall, these results suggest that quality-of-life measures may be less sensitive to short-term interventions, in contrast with the consistent improvements observed in functional outcomes. Longer follow-up periods and standardized scales are needed to clarify the impact of prehabilitation on patient-centered outcomes.

### 4.6. Proposed Clinical Model of Prehabilitation in Cardiac Surgery and Future Perspectives

This review supports exercise, when properly prescribed and supervised, as a potentially effective preoperative intervention, particularly in frail patients. Prehabilitation improved functional capacity, reduced respiratory complications, and shortened hospital stays, yet remains underused in clinical practice.

Many current programs are short, low intensity and poorly individualized, which may explain the limited effects on quality of life or psychological outcomes. Standardization in cardiac surgery prehabilitation remains lacking [31], reinforcing the need for structured interventions tailored to physical condition and frailty, integrating aerobic, resistance and inspiratory muscle training.

We propose a clinical model of prehabilitation structured around three pillars (Table 3): (i) moderate-intensity aerobic training on a cycle ergometer [32]; (ii) progressive resistance training targeting large muscle groups to improve functionality and reduce dynapenia; and (iii) inspiratory muscle training with incentive devices to reduce pulmonary complications. Program design should be grounded in exercise physiology principles. Individualization is essential, particularly for patients with comorbidities, and rehabilitation physicians are central to adapting loads and progression. Progressive overload ensures adaptation while preventing both under- and over-stimulation. Gradual progression supports safe improvement, and training specificity aligns exercise with functional demands.

Based on current evidence and physiological principles, programs lasting at least two weeks may be needed to achieve meaningful adaptations [33], with an optimal range of 4–8 weeks to maximize functional and respiratory benefits. From a clinical perspective, aerobic training should be preferentially delivered on a cycle ergometer rather than a treadmill, given its greater safety, stability, and reduced joint loading—factors particularly relevant for older and frail patients [34]. MICT remains the most feasible and broadly applicable modality, while HIIT may be considered for selected patients with good baseline tolerance and adequate supervision, as it can induce rapid cardiorespiratory gains within limited preoperative timeframes [35].

Resistance training should prioritize functional, multi-joint exercises targeting major lower-limb and trunk muscle groups, using simple and accessible equipment such as elastic bands, dumbbells or bodyweight. In our view, prescribing loads at 50–60% of 1RM with 2–3 sets of 8–12 repetitions and applying progressive overload of 5–10% based on individual tolerance represents a pragmatic and safe strategy [36].

Finally, inspiratory muscle training, though inconsistently reported in the included studies, should be structured according to MIP. We recommend initiating at 30–50% of baseline MIP, with weekly progression of 5–10%, and performing at least 2 sets of 30 breaths on 5 days per week [37,38]. This structured approach could maximize respiratory benefits and potentially reduce postoperative pulmonary complications.

Notably, the proposed model represents a conceptual framework synthesized from the current evidence rather than a validated clinical protocol.

Our study highlights the therapeutic role of structured exercise in cardiac surgery. While prehabilitation has been extensively studied in oncology and orthopedics, its application in cardiac surgery remains relatively recent. Few trials have combined aerobic, resistance, and inspiratory training, yet our findings demonstrate consistent gains in functional capacity and reductions in pulmonary complications and hospital stay, particularly among frail or dynapenic patients.

Extending well-established concepts from cardiovascular rehabilitation to the perioperative context bridges sports science and clinical cardiology, offering a framework for multimodal, individualized interventions. Multidisciplinary collaboration—involving rehabilitation physicians, physiotherapists, and exercise scientists—will be key to translating these programs into clinical practice.

### 4.7. Strengths and Limitations

The present review provides the most comprehensive and up-to-date synthesis of exercise-based prehabilitation in cardiac surgery, incorporating randomized evidence published up to 2025. Beyond confirming the efficacy of prehabilitation, it explores clinically meaningful moderators through meta-regression analyses and sensitivity testing, offering a more nuanced understanding of program duration, respiratory training and patient characteristics. It also expands the evidence base by addressing frailty, sarcopenia and dynapenia—factors often overlooked in previous reviews—and integrates these findings into a structured clinical model that can guide perioperative practice.

In terms of limitations, the heterogeneity of surgical procedures, program duration, intensity and components limits the generalizability of the findings. Some included trials had small sample sizes, reducing statistical power to detect rare outcomes. Only nine studies met the inclusion criteria, yet they represent all available evidence on the topic. Generalization to other populations with differing baseline characteristics might be limited. Differences in assessment tools for quality of life and functional status may also account for nonsignificant results. Moreover, heterogeneity in the definitions of postoperative complications may have influenced the pooled estimates and highlights the importance of adopting standardized outcome criteria to improve comparability and clinical interpretation across trials.

### 4.8. Future Directions

Future research should standardize exercise protocols, incorporate robust measures of frailty and patient-reported outcomes and confirm long-term benefits in multicenter trials. Prehabilitation thus represents a promising frontier where sports and exercise medicine can meaningfully improve surgical outcomes.

Prehabilitation should no longer be regarded merely as an optional adjunct but rather as a promising and increasingly relevant component of perioperative care.

## 5. Conclusions

Exercise-based prehabilitation is a safe and promising strategy for patients undergoing cardiac surgery. It is consistently associated with improvements in functional capacity and with lower rates of respiratory complications and shorter hospital stays, with the greatest benefits observed in frail patients and in those with reduced muscle strength.

Multimodal programs that combine aerobic training, progressive resistance exercise and inspiratory muscle training appear most effective. Programs lasting at least four weeks are advisable to maximize physiological adaptation and preoperative readiness.

An important consideration is the frequent confusion between sarcopenia (loss of muscle mass) and dynapenia (loss of muscle strength). Most studies assessed strength using handgrip testing, which reflects dynapenia rather than true sarcopenia. Clarifying this distinction is essential, as both conditions are clinically relevant and should be targeted as modifiable risk factors within prehabilitation programs.

These findings support the systematic integration of exercise-based prehabilitation into perioperative care, while underscoring the need for standardized protocols and further multicenter trials to confirm long-term benefits and cost-effectiveness.

## Figures and Tables

**Table 1:** General characteristics of the studies included.

Authors	Sample Size (n)	Country	Age (Years)	Sex (% Male)	CV Risk Factors	Surgery Type	Main Outcomes
Herdy et al. (2008) [4]	56	Brazil	61 ± 10	72%	Smoking	CABG	Improvement in 6MWT after prehabilitation
Rosenfeldt et al. (2011) [5]	117	Australia	65.2 ± 6	74%	DM	CABG (61%), VD (39%)	Need for longer-lasting prehabilitation programs to impact quality of life
Sawatzky et al. (2014) [6]	17	Canada	64 ± 8	80%	HTN, DM, DLP	CABG	Significant improvement in functional capacity after prehabilitation (6MWT, gait speed test), as well as greater adherence to physical exercise. No differences in postoperative complications
Waite et al. (2017) [7]	20	England	>65	Not reported	Not reported	CABG and VD	Significant improvement in functional capacity (6MWT, SPPB) and frailty status (CFS reduction)
Steinmetz et al. (2020) [8]	203	Germany	67.1 ± 8	Not reported	HTN, DM, DLP, smoking	CABG	Significant improvement in functional capacity and quality of life after prehabilitation (6MWT, TUGT)
Akowuah et al. (2023) [9]	180	England	50–70	82%	Not reported	CABG (56.7%), AVD (41.7%), MVD (15.4%), TVD (1.1%), aortic pathology (10%), others (5%)	No differences in functional capacity improvement after prehabilitation (only in those with dynapenia). Improvement observed in inspiratory muscle strength
López-Hernández et al. (2024) [10]	68	Spain	71 ± 10	69%	Not reported	Aortic VD (58.8%), mitral VD (42.2%)	Significant improvement in functional capacity in 6MWT, handgrip strength test, and endurance time (ET) on cycle ergometer after prehabilitation
Sahar et al. (2024) [11]	74	Pakistan	54 ± 6	83%	DM, smoking	CABG	Improved surgical outcomes after strength training. No significant differences in frailty status
Yau et al. (2025) [12]	138	China	64 ± 10	69%	DM	CABG (53%), VD (47%)	Improvement in functional capacity (6MWT) without an increase in adverse events after surgery

AVD: Aortic Valve Disease; CABG: Coronary Artery Bypass Grafting; CFS: Clinical Frailty Scale; CV: Cardiovascular; DLP: Dyslipidemia; DM: Diabetes Mellitus; HTN: Hypertension; MVD: Mitral Valve Disease; SPPB: Short Physical Performance Battery; TUGT: Time Up and Go test; TVD: Tricuspid Valve Disease; VD: Valvular Disease; 6MWT: 6 min walk test.

**Table 2:** Summary of meta-regression analyses evaluating moderators of functional improvement as assessed by the 6 min walk test.

Model	Participants	β Coefficient	95% CI	Adjusted R^2^	*p* Value
Program duration (weeks)	873	14.76	[−9.5, 39]	0.07	0.287
Age (years)	873	−9.26	[−20.6, 2.1]	0.21	0.169
Respiratory training (yes vs. no)	873	−90.25	[−176.9, −3.6]	0.352	0.094

**Table 3:** Proposed clinical prehabilitation model for cardiac surgery.

	Equipment	Intensity	Duration	Progression	Exercises
**Aerobic**	Cycle ergometer	Moderate - VO_2_max: 40–69% - HRmax: 55–74% - HRR: 40–69%	25–30 min	Increase time by 2–3 min per week	MICT (standard) or HIIT (for selected patients with good tolerance)
**Strength**	Dumbbells, ankle weights, elastic bands	50–60% 1RM	2–3 sets of 8–12 reps	Increase load by 5–10% every 1–2 weeks	- Lower limbs: Squats, step-up, quadriceps extension (banded), glute bridge - Upper limbs: triceps kickback, biceps curl, banded row, banded press
**Respiratory**	Incentive spirometer	30–50% of initial MIP	3 sets of 10 deep inspirations, twice daily	Increase resistance by 5–10% per week	Maximal inspirations against resistance, diaphragmatic breathing with resistance, lower costal expansions

1RM: one-repetition maximum; HIIT: High-Intensity Interval Training; HRmax: Maximal heart rate; HRR: Heart rate reserve; MICT: Moderate-Intensity Continuous Training; MIP: Maximal inspiratory pressure; VO_2_max: Maximal oxygen uptake.

## Data Availability

The data presented in this study are available upon reasonable request to the corresponding author.

## Supplementary Materials

The following supporting information can be downloaded at: https://www.mdpi.com/article/10.3390/jcm14228195/s1, Table S1: Checklist PRISMA 2020 guidelines; Table S2: Search equations and review process; Table S3: Domain-based assessment of the risk of bias of each study included according to the Cochrane Risk of Bias Tool (RoB 2); Table S4: Evaluation of the Grading of Recommendations Assessment, Development and Evaluation (GRADE scale) of the outcomes of the meta-analysis; Table S5: Characteristics of prehabilitation programs; and Figure S1: Funnel plots assessing publication bias for the primary and secondary outcomes [39].
