# Supplementary material for: Exercise-Based Prehabilitation Before Cardiac Surgery: A Systematic Review, Meta-Analysis, Meta-Regression, and Proposal for a Clinical Implementation Model
Source: J Clin Med. 2025 Nov 19;14(22):8195. doi: 10.3390/jcm14228195 (PMC12653149; doi:10.3390/jcm14228195)

SUPPLEMENTARY MATERIAL

Supplementary Table S1. Checklist PRISMA 2020 guidelines.

SECTION	ITEM	PRISMA-ScR CHECKLIST ITEM	REPORTED ON PAGE #
**TITLE**			
Title	1	Identify the report as a scoping review.	1
**ABSTRACT**			
Structured summary	2	Provide a structured summary that includes (as applicable): background, objectives, eligibility criteria, sources of evidence, charting methods, results, and conclusions that relate to the review questions and objectives.	1
**INTRODUCTION**			
Rationale	3	Describe the rationale for the review in the context of what is already known. Explain why the review questions/objectives lend themselves to a scoping review approach.	2
Objectives	4	Provide an explicit statement of the questions and objectives being addressed with reference to their key elements (e.g., population or participants, concepts, and context) or other relevant key elements used to conceptualize the review questions and/or objectives.	2
**METHODS**			
Protocol and registration	5	Indicate whether a review protocol exists; state if and where it can be accessed (e.g., a Web address); and if available, provide registration information, including the registration number.	2
Eligibility criteria	6	Specify characteristics of the sources of evidence used as eligibility criteria (e.g., years considered, language, and publication status), and provide a rationale.	2
Information sources*	7	Describe all information sources in the search (e.g., databases with dates of coverage and contact with authors to identify additional sources), as well as the date the most recent search was executed.	2
Search	8	Present the full electronic search strategy for at least 1 database, including any limits used, such that it could be repeated.	2
Selection of sources of evidence†	9	State the process for selecting sources of evidence (i.e., screening and eligibility) included in the scoping review.	2
Data charting process‡	10	Describe the methods of charting data from the included sources of evidence (e.g., calibrated forms or forms that have been tested by the team before their use, and whether data charting was done independently or in duplicate) and any processes for obtaining and confirming data from investigators.	2
Data items	11	List and define all variables for which data were sought and any assumptions and simplifications made.	3
Critical appraisal of individual	12	If done, provide a rationale for conducting a critical appraisal of included sources of	3

SECTION	ITEM	PRISMA-ScR CHECKLIST ITEM	REPORTED ON PAGE #
sources of evidence§		evidence; describe the methods used and how this information was used in any data synthesis (if appropriate).	
Synthesis of results	13	Describe the methods of handling and summarizing the data that were charted.	3
**RESULTS**			
Selection of sources of evidence	14	Give numbers of sources of evidence screened, assessed for eligibility, and included in the review, with reasons for exclusions at each stage, ideally using a flow diagram.	4
Characteristics of sources of evidence	15	For each source of evidence, present characteristics for which data were charted and provide the citations.	5
Critical appraisal within sources of evidence	16	If done, present data on critical appraisal of included sources of evidence (see item 12).	3
Results of individual sources of evidence	17	For each included source of evidence, present the relevant data that were charted that relate to the review questions and objectives.	6
Synthesis of results	18	Summarize and/or present the charting results as they relate to the review questions and objectives.	8
**DISCUSSION**			
Summary of evidence	19	Summarize the main results (including an overview of concepts, themes, and types of evidence available), link to the review questions and objectives, and consider the relevance to key groups.	14
Limitations	20	Discuss the limitations of the scoping review process.	17
Conclusions	21	Provide a general interpretation of the results with respect to the review questions and objectives, as well as potential implications and/or next steps.	19
**FUNDING**			
Funding	22	Describe sources of funding for the included sources of evidence, as well as sources of funding for the scoping review. Describe the role of the funders of the scoping review.	19

Supplementary Table S2. Search equations and review process.

Database	Search strategies	Number of articles
**PUBMED**	(((((prehabilitation) OR (preoperative exercise)) AND (cardiac surgery)) OR (valv*) OR (coronary artery bypass graft surgery) AND (functional capacity) NOT (transcatheter))) NOT (cancer).<br>Filters: Randomised controlled trial; Publication date to 2005:2025.	214
**Cochrane Library**	((prehabilitation:ti,ab,kw OR "preoperative exercise":ti,ab,kw OR "preoperative training":ti,ab,kw OR "exercise therapy":ti,ab,kw) AND ("cardiac surgery":ti,ab,kw OR "heart surgery":ti,ab,kw OR valv*:ti,ab,kw OR "coronary artery bypass graft":ti,ab,kw) AND ("functional capacity":ti,ab,kw OR "exercise capacity":ti,ab,kw)) NOT (transcatheter:ti,ab,kw OR cancer:ti,ab,kw)<br>Filters: Date published March 2005 – March 2025	64
**PEDro**	a. prehabilitation AND cardiac surgery AND functional	4
	b. prehabilitation AND cardiac surgery AND functional capacity	3
**LILACS**	(prehabilitation) OR (preoperative exercise) AND (cardiac surgery) AND (functional capacity)	45

Supplementary Table S3. Domain-based assessment of the risk of bias of each study included according to the Cochrane Risk of Bias Tool (RoB 2).

**STUDY (year)**	Random sequence generation (selection bias)	Allocation concealment (selection bias)	Blinding of participants and personnel (performance bias)	Blinding of outcome assessment (detection bias)	Incomplete outcome data (attrition bias)	Selective reporting (reporting bias)	Other bias
Herdy (2008) [4]	+	?	–	?	+	+	+
Rosenfeldt (2011) [5]	?	?	–	?	+	+	?
Sawatzky 2014) [6]	+	?	–	?	+	+	+
Waite (2017) [7]	?	?	–	?	+	+	?
Steinmetz 2020) [8]	+	+	–	+	+	+	+
Akowuah (2023) [9]	?	?	–	?	+	+	?
López-Hernández (2024) [10]	+	+	–	+	+	+	+
Sahar (2024) [11]	+	?	–	?	+	+	+
Yau (2025) [12]	+	+	–	+	+	+	+

Green circle with "+": Low risk of bias; Yellow circle with "?": Some concerns; Red circle with "–": High risk of bias.

Supplementary Table S4. Evaluation of the Grading of Recommendations Assessment, Development and Evaluation (GRADE scale) of the outcomes of the meta-analysis.

**Outcome:** Functional Capacity assessed by the 6-minute walk test.			
**GRADE criteria**	**Rating**	**Reasons**	**Quality**
**Study design**	RCT (starts at high quality)	All included studies are RCTs.	
**Risk of Bias**	No.	Although some of the items of the RoB Scale were evaluated as "some concerns" and the blinding of participants as "high risk of bias", these are inherent methodological characteristics of exercise interventions, where patients cannot be unaware of being exercising. The rest of the items presented no serious risk of bias.	
**Inconsistency**	Serious (–1).	Considerable heterogeneity was found in initial analysis (I^2 = 76%), yet subgroup analyses based on duration and inclusion of inspiratory muscle training lowered it to 0% and 45%, respectively. However, a point is reduced from total quality since moderate heterogeneity remains.	Moderate (●●●○)
**Indirectness**	No.	PICO correspond to that of the present meta-analysis.	
**Imprecision**	No.	Narrow confidence intervals that do not cross the clinical relevance threshold and significant results with low *P*.	
**Publication Bias**	Undetected.	Forest plots examined with undetected publication bias.	
**Other factors**	No.		

**Outcome:** Post-Operative Complications.			
**GRADE criteria**	**Rating**	**Reasons**	**Quality**
**Study design**	RCT (starts at high quality)	All included studies are RCTs.	Arrhythmias: low (●●○○)<br>Atelectasis: high (●●●●)<br>Extubation: low (●●○○)<br>Hospital stay: very low (●○○○)<br>ICU stay: moderate (●●●○)
**Risk of Bias**	No.	Although some of the items of the RoB Scale were evaluated as "some concerns" and the blinding of participants as "high risk of bias", these are inherent methodological characteristics of exercise interventions, where patients cannot be unaware of being exercising. The rest of the items presented no serious risk of bias.	
**Inconsistency**	Arrhythmias: serious (–1).<br>Atelectasis: no.<br>Extubation: serious (–1).<br>Hospital: very serious (–2).<br>ICU stay: no.	Arrhythmias: $I^2 = 62\%$<br>Atelectasis: $I^2 = 0\%$<br>Time to Extubation: $I^2 = 50\%$<br>Hospital length of stay: $I^2 = 84\%$<br>ICU length of stay: $I^2 = 0\%$	
**Indirectness**	No.	PICO correspond to that of the present meta-analysis.	
**Imprecision**	Arrhythmias: serious (–1).<br>Atelectasis: no.<br>Extubation: serious (–1).<br>Hospital: serious (–1).<br>ICU stay: serious (–1).	Arrhythmias: confidence interval (CI) includes benefits and no effect.<br>Atelectasis: narrow CI showing significant benefits.<br>Time to Extubation: CI encompasses benefits and no effect.<br>Hospital length of stay: CI encompasses benefits and no effect.<br>ICU length of stay: CI encompasses benefits and no effect.	
**Publication Bias**	Undetected.	Forest plots examined with undetected publication bias.	
**Other factors**	Atelectasis: large effect (+1).	Atelectasis showed a large effect (OR < 0.5) with a narrow CI and significant results ($P < 0.05$) in the absence of heterogeneity ($I^2 = 0\%$).	

Supplementary Table S5. Characteristics of prehabilitation programs.

Authors (Year)	Sample size (n)	Exercise-based prehabilitation	Characteristics	Equipment	Duration	Intensity	Control group
**Herdy (2008) [4]**	56	Strength training + respiratory muscles	- Strength: Not specified<br>- Respiratory: Not specified	Inspiratory device	5 days	Not specified	Not intervention
**Rosenfeldt (2011) [5]**	117	Aerobic exercise + stress reduction / management techniques	- Aerobic: 3 x 15 min constant (5 min rest)<br>- Stress: Meditation, Deep breathing, coping strategies	Cycle ergometer, treadmill, arm ergometer	2 weeks (2 sessions per week of 60 min each)	Light (60% HRmax)	Not intervention
**Sawatzky (2014) [6]**	17	Aerobic exercise + strength training + stretching	- Aerobic: Not specified<br>- Strength: Not specified<br>- Stretching: Not specified	Cycle ergometer, treadmill, elastic bands	4 weeks (2 sessions per week of 60 min each)	Moderate (85% VO_2max)	Not intervention
**Waite (2017) [7]**	20	Otago exercise program	- Balance: Single-leg stance, heel/toe walking, tandem gait<br>- Strength: 3 x 10 reps (gluteals, calves, quadriceps, triceps, biceps, back)	Elastic bands (individualized)	4-6 weeks (3 session per week 20-40 min each)	Light (Borg scale 4-5)	Not intervention
**Steinmetz (2020) [8]**	203	Aerobic exercise + balance + breathing techniques	- Aerobic: 2 x 20min constant (daily progressive increase)<br>- Balance: Not specified (chair-based)<br>- Respiratory: 1 x 15 min (not specified)	Cycle ergometer	2 weeks (3 sessions per week of 60 min each)	Light (70% HRmax)	Not intervention
**Akowuah (2023) [9]**	180	Strength training + respiratory muscles	- Strength: Not specified<br>- Respiratory: Not specified	Inspiratory device	4 weeks (2 sessions per week of 60 min each)	Not specified	Lifestyle advice and healthy habits counselling

Supplementary Table S5. Characteristics of prehabilitation programs (cont.)

Authors (Year)	Sample size (n)	Exercise-based prehabilitation	Characteristics	Equipment	Duration	Intensity	Control group
**López-Hernández (2024) [10]**	68	Aerobic exercise + strength + respiratory muscles	- Aerobic: HIIT (2 min at 70-80% HRmax, 2 min at 40%)<br>- Strength: 2-3 x 8-15 reps (exercises not specified)<br>- Respiratory: 1-2 x 10-15 reps (thoracic expansions, diaphragmatic breathing, deep inspirations)	Cycle ergometer, volumetric incentive spirometer	4-6 weeks (2 sessions per week of 60 min each)	HIIT (70-80% HRmax with active rest at 40% HRmax)	Nutritional counselling, physical activity promotion, smoking cessation advice
**Sahar (2024) [11]**	74	Strength	1-3 x 10-15 reps at 40-50% 1RM: Shoulder press, biceps curl, knee raises, quadriceps extension, hamstring curl	Dumbbells, weights	8 weeks (3 sessions per week)	Light (40-50% 1RM)	Inspiratory muscle training and cough assistance
**Yau (2025) [12]**	138	Aerobic exercise + strength	- Aerobic: 20-60 min constant (exercise not specified)<br>- Strength: 1-3 x 10-15 reps (large lower/upper limb muscles group not specified)	Dumbbells, weights	6-8 weeks (2 sessions per week)	Light (40-80% VO_2max)	Pre- and postoperative surgical counselling

1RM: One-repetition maximum; HIIT: High-intensity Interval training; HRmax: Maximum heart rate; Reps: Repetitions; VO2max: Maximal oxygen uptake.

Supplementary Figure S1. Funnel plots assessing publication bias for the primary and secondary outcomes: (A) 6MWT, (B) Hospital stay, (C) ICU stay, (D) incidence of arrhythmias, (E) incidence of atelectasis, (F) Time to Extubation.

A

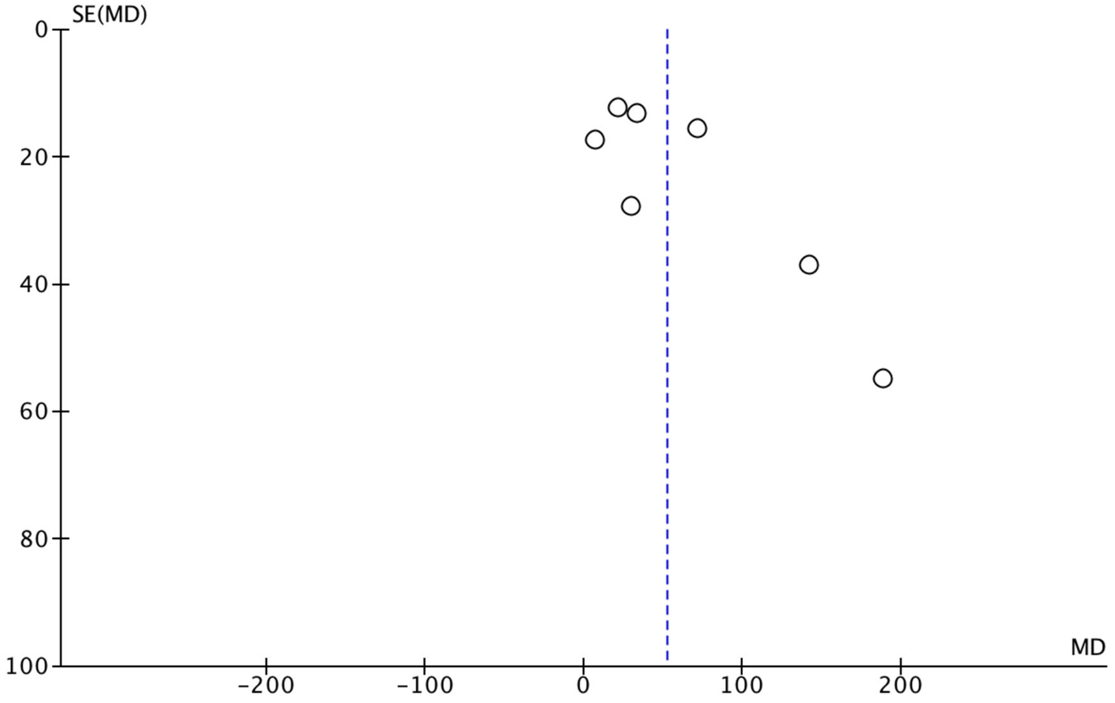

B

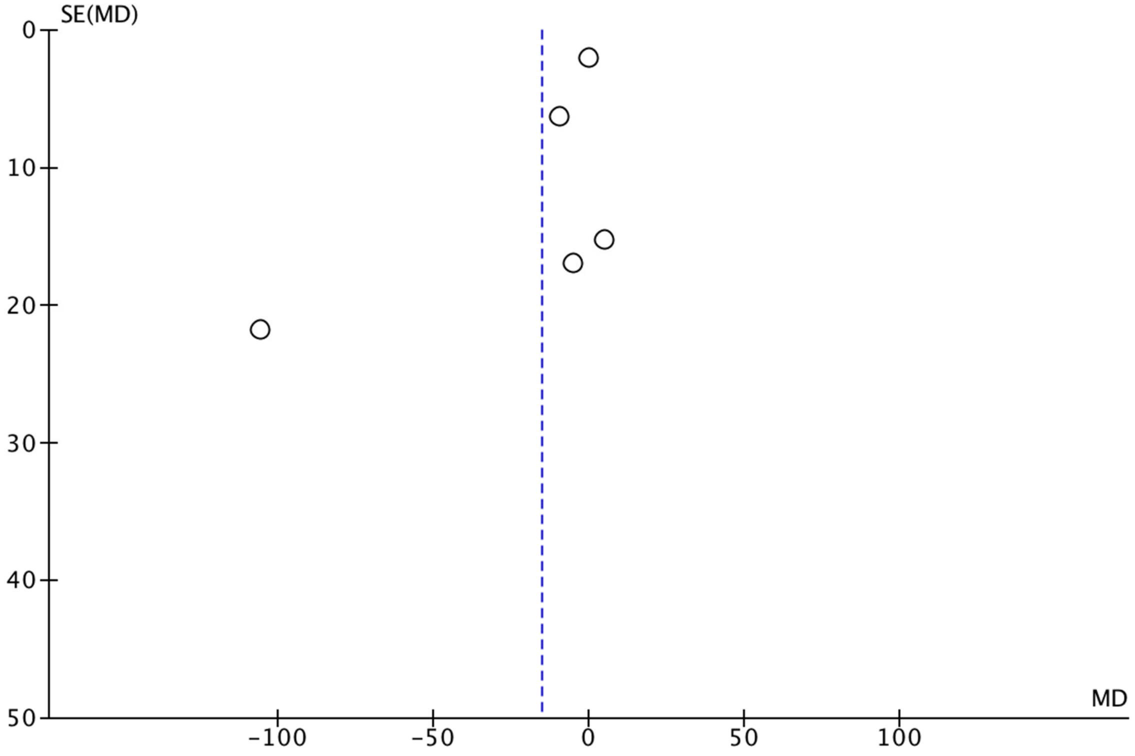

C

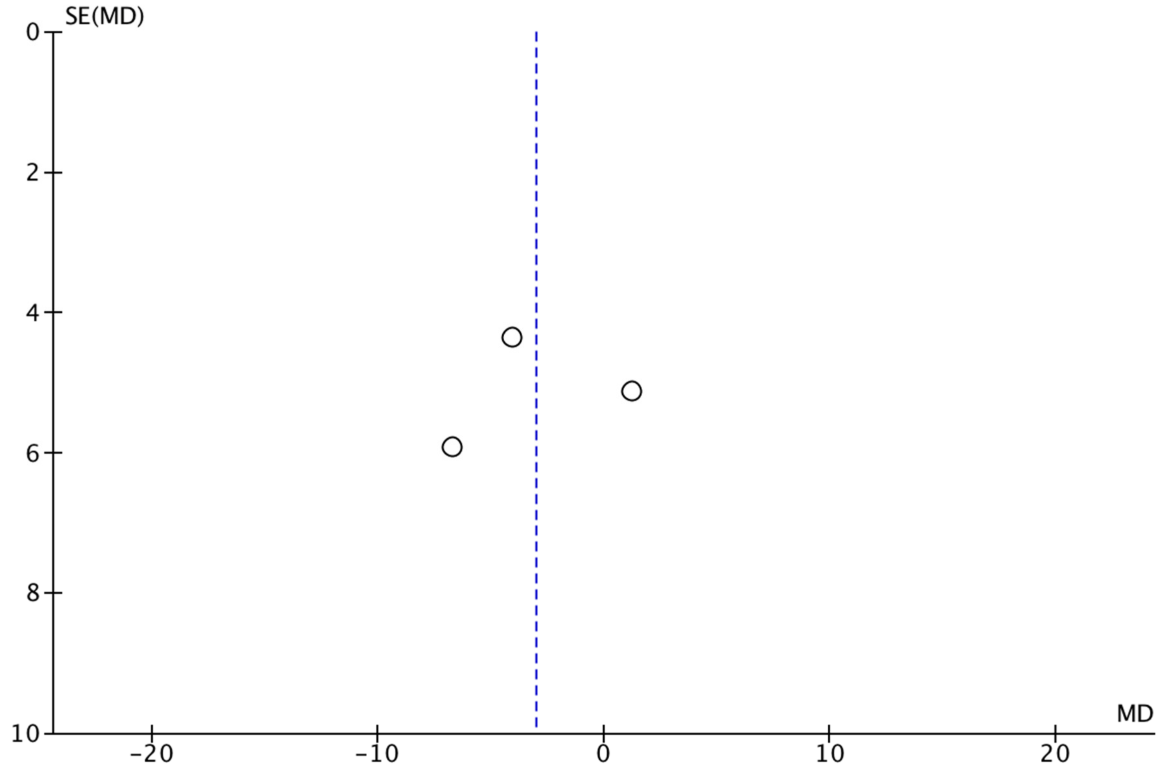

D

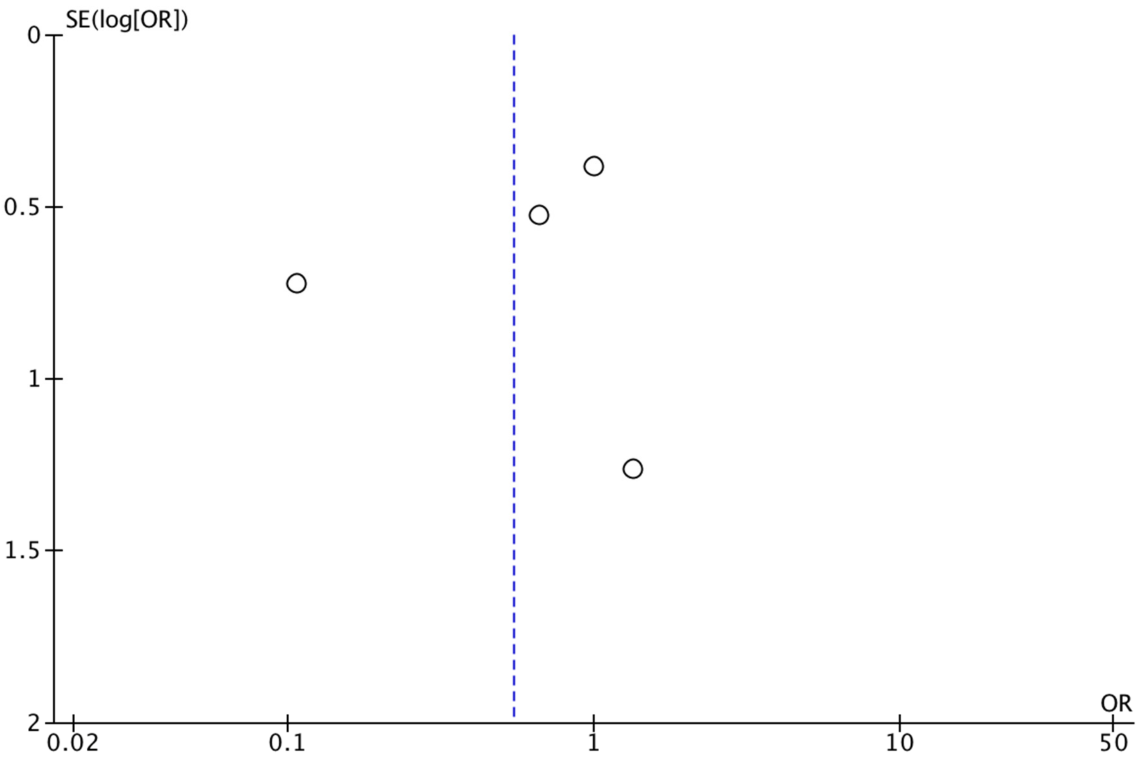

E

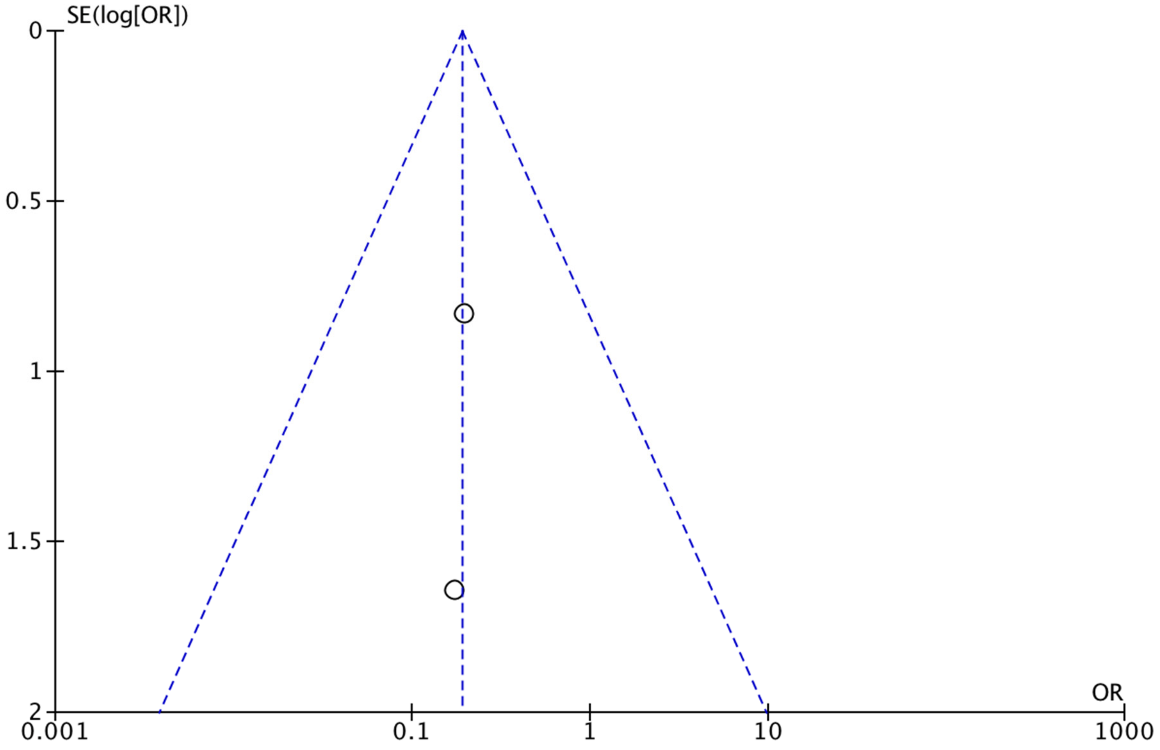

F

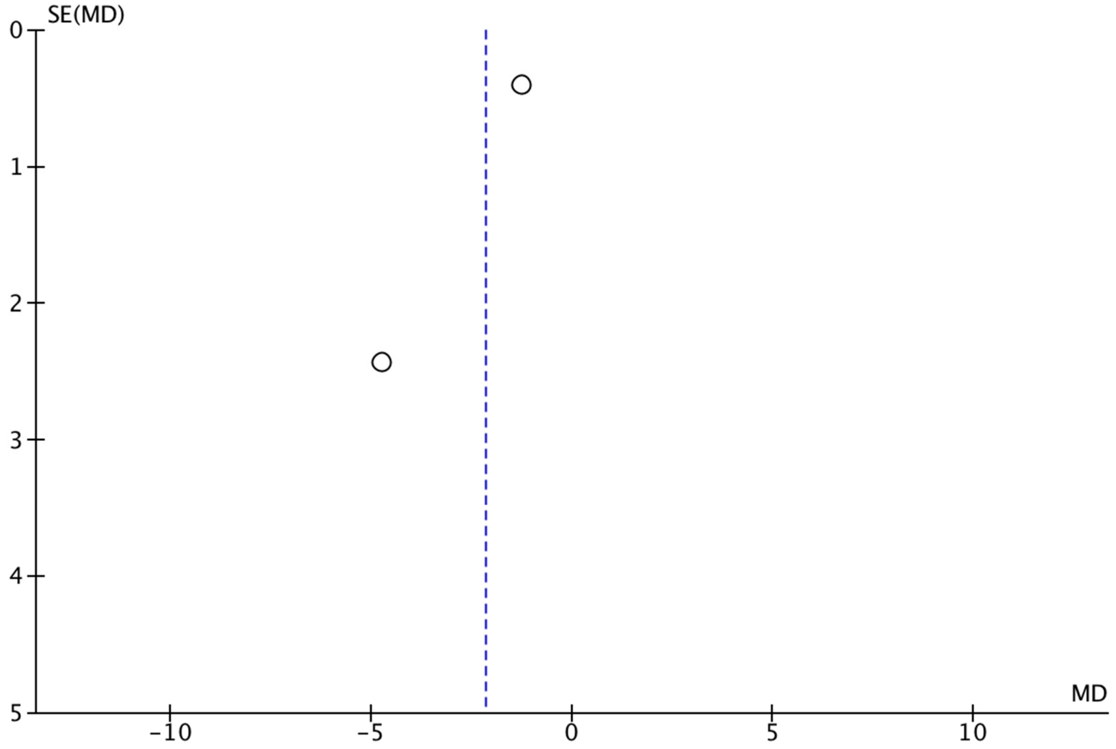

Supplement: Supplementary file 1 [file jcm-14-08195-s001.zip › jcm-3964144-supplementary.pdf]
